# Optogenetic Control of Protein Function: From Intracellular Processes to Tissue Morphogenesis

**DOI:** 10.1016/j.tcb.2016.09.006

**Published:** 2016-11

**Authors:** Giorgia Guglielmi, Henning Johannes Falk, Stefano De Renzis

**Affiliations:** 1 European Molecular Biology Laboratory (EMBL), Meyerhofstrasse 1, 69117 Heidelberg, Germany

## Abstract

Optogenetics is an emerging and powerful technique that allows the control of protein activity with light. The possibility of inhibiting or stimulating protein activity with the spatial and temporal precision of a pulse of laser light is opening new frontiers for the investigation of developmental pathways and cell biological bases underlying organismal development. With this powerful technique in hand, it will be possible to address old and novel questions about how cells, tissues, and organisms form. In this review, we focus on the applications of existing optogenetic tools for addressing issues in animal morphogenesis.

## Trends

Optogenetics allows the manipulation of gene expression and cell behaviors in individual cells or group of cells in the context of intact organisms.

Precise spatiotemporal and quantitative perturbation of signaling systems using optogenetics provides a powerful new approach to study morphogenesis of multicellular systems.

The possibility to combine optogenetics with genetically encoded biosensors (e.g., tension sensors) or chemical probes enables the perturbation and monitoring of biochemical reactions of interest *in vivo*.

By allowing the control of key behaviors, such as cell motility, polarity, and proliferation, optogenetics will be instrumental for characterizing the mechanisms underlying organ regeneration and cancer growth/invasion in living organisms.

## Interrogating Morphogenesis

Morphogenesis, the shaping of living cells and tissues, is a formidably complex process that involves the concerted action of thousands of molecules at specific locations and at defined times. Across scales, from single-celled organisms to complex animals comprising tens of thousands of cells, the action of these molecules results in changes in cell behavior, which ultimately determine the function of the cell. For example, the unicellular yeast-like fungus *Saccharomyces cerevisiae* can either form chains of elongated cells, or reproduce asexually by reorienting its actin cytoskeleton to form a polarized bud, depending on the availability of specific nutrients 1, 2. Similarly, in multicellular organisms, individual cells, such as neurons or intestinal epithelial cells, acquire different shapes that are adapted to perform a specific physiological function. On a larger scale, morphological remodeling is driven by changes in cell behavior in single cells or group of cells. For example, during embryonic development, tissue and organ growth are often initiated by localized changes in cell shape, motility, and proliferation rates. Therefore, understanding morphogenesis requires, on the one hand, an understanding of the way cells restructure their internal contents, and on the other, of how cells coordinate their behavior to build macroscopic structures.

Bridging the subcellular and supracellular scales at the functional level requires tools that allow the control of protein activity and cell behavior with high spatiotemporal precision. Standard genetic approaches – knockdown, knockout, overexpression, and mutation – have broad effects on the organism and act on long timescales. Chemical approaches can rapidly switch on or off the function of specific proteins, but they do not allow spatial control. In the past years, laser dissection has become a popular tool to perturb single cells during morphogenesis 3, 4, 5, 6. This technique enables the severing of actomyosin filaments or microtubules with high intensity lasers, prompting localized and fast responses. However, its use is limited to the destruction of specific cytoskeletal structures and does not enable the modulation of other cellular features.

**Figure 1:**
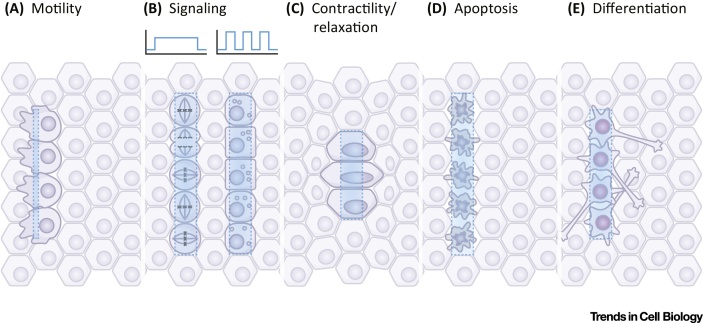
Optogenetic Applications in Cell and Tissue Biology. Each panel represents a cell sheet in a resting state. Blue stripes indicate light patterns triggering optogenetic activation of specific behaviors in a subgroup of cells. (A) Stimulation of protrusion formation and directed motility. (B) Modulation of signal strength and dynamics. The duration and frequency of the light signal is schematized as light intensity versus time of a continuous (left) and a pulsed (right) input. Continuous activation of specific signaling pathways stimulates proliferation (illuminated cells, left) while pulsed activation differentiation (illuminated cells, right). See text for details. (C) Light-mediated modulation of the actomyosin cytoskeleton can be used to stimulate or inhibit cell contractility. (D) Programmed cell death can be triggered at will using spatiotemporal patterns of optogenetic activation. (E) Optogenetic control of gene expression enables the temporally precise initiation of cell differentiation in individual cells and tissues, as well as entire organisms.

More recently, advances in optogenetics have offered new means to modulate protein function with unprecedented spatiotemporal precision. Light-sensitive modules have been used to probe signaling cascades, stimulate gene expression, alter cell polarity, and trigger cell motility [7] (Figure 1). In addition, optogenetics has been used to modulate specific morphogenetic events in intact organisms. Here, we review the optogenetic approaches that have been used to control both cell and tissue behaviors, and the questions that they have allowed us to tackle. We discuss the potential of optogenetics for addressing questions related to how different shapes arise from a stereotyped set of molecular and cellular activities, and how changes in behavior in individual cells influence neighboring cells and global tissue remodeling.

## Optogenetic Modulation of Molecular and Cellular Processes Driving Morphogenesis

**Figure 2:**
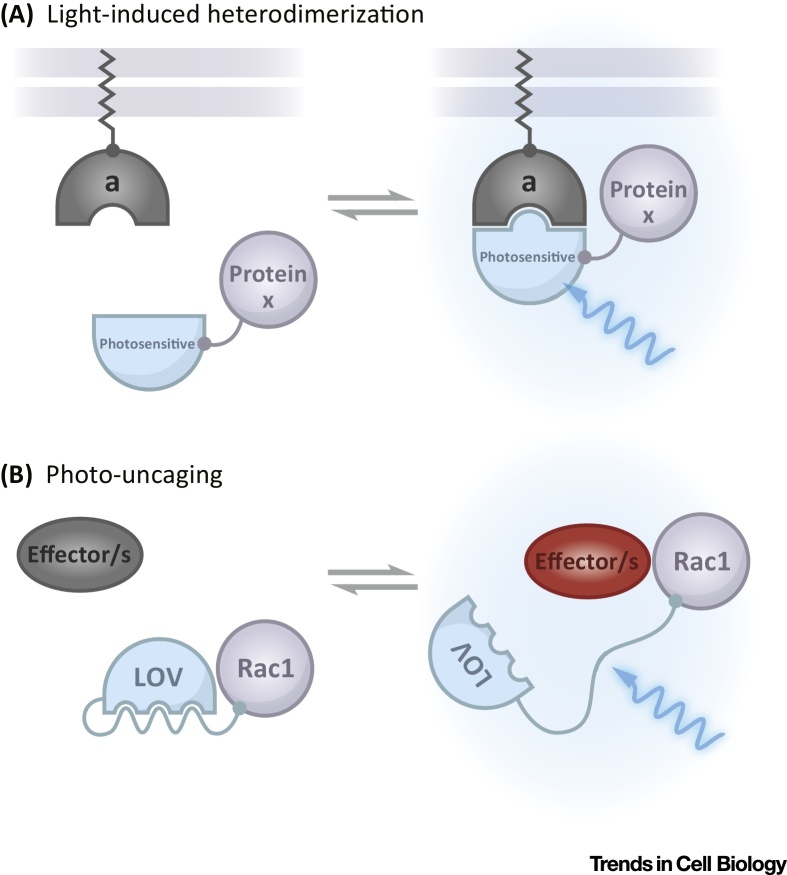
Schematic Illustration of Light-Sensitive Protein Heterodimerization and Protein Photo-Caging Methods. Upper panel represents a generic illustration of how light-sensitive protein heterodimerization systems function. Lower panel illustrates the specific case of how the phototropin1 LOV2 photosensitive protein domain from *Avena sativa* has been used to control the small GTPase Rac1 with light [10]. (A) A cytosolic protein of interest (protein x) is tagged with a photosensitive protein domain that interacts with its binding partner (a) upon light illumination (right panel). By anchoring component (a) to a specific membrane compartment (e.g., the plasma membrane) it is possible to recruit protein x to that compartment upon light illumination (left panel). See Table 1 for more details. (B) The small GTPase Rac1 has been tagged with the phototropin1 LOV2 domain from *Avena sativa*, which sterically inhibits interaction of Rac1 with its downstream effectors (left panel). Upon a pulse of blue light illumination (458 or 473 nm), unwinding of a helix linking LOV2 to Rac1 frees Rac1 from inhibition, allowing it to interact with its binding partner, thus restoring protein activity in a spatiotemporally controlled manner.

During animal development, simple cell layers are transformed into complex tissues and organs through different strategies. Cells can move individually or as collectives, and migrate to different places. Tissue monolayers can assume complex structures by folding, elongating, or altering cell number via proliferation or programmed cell death. All these processes are highly dependent on the timing, location, and intensity of developmental inputs, and rely on the coordination between polarity cues, cytoskeletal and membrane dynamics, cell adhesion, signaling, and gene expression. Below we describe some of the morphogenetic events that sculpt the embryo, and the optogenetic tools that have been developed and applied – or have the potential to be applied – for addressing questions in cell and developmental biology (Table 1 and Figure 2).

### Epithelial Morphogenesis and Tissue Invagination

Epithelia are sheets of cells that separate different compartments of the body, and constitute the functional unit of internal organs such as liver, kidneys, and the digestive and respiratory systems. Epithelial cells are highly polarized along their apical–basal axis and tightly interconnected through adherens junctions (AJs), cell–cell contacts that run around each cell as a ‘belt’. The main component of AJs is E-cadherin, a calcium-dependent transmembrane protein whose cytoplasmic portion is linked, via catenins and other adaptors, to the actomyosin cytoskeleton. By coupling the cytoskeleton of neighboring cells, AJs transmit the subcellular tension generated by the contractile activity of myosin on actin filaments across tissues 8, 9. Due to the tight intercellular connections, cell movements within epithelia are limited. Therefore, remodeling of epithelial tissues during embryonic development relies mainly on exchange of cell neighbors, tissue stretching, flattening, and folding. All these processes depend on changes in cell shape and/or cell–cell contacts, and require precise spatiotemporal coordination of actomyosin contractility.

Despite advances in live imaging, which allow the visualization of epithelial morphogenesis in great detail, several key questions remain difficult to address with current methodologies. In particular, it is unclear how actomyosin networks of distinct molecular composition are assembled at specific sites, and how the resulting contractile properties (e.g., pulsatile behavior) drive the different modes of epithelial rearrangement observed *in vivo*. Furthermore, little is known about the impact of changes in cortical tension on other intracellular processes controlling cell shape, such as microtubule and membrane dynamics, and vice versa. At the tissue level, outstanding questions relate to how cells coordinate their behavior, respond to changes in tissue tension (**mechanosensation**, see Glossary), and how geometrical constraints impact on tissue and organ shape. Light-sensitive protein heterodimerization systems provide particularly useful tools to address these questions in living organisms. These dual-component systems are based on the interaction, upon light exposure, between a light-sensitive protein domain and its binding partner. By tagging a protein of interest with one of these two modules and anchoring the other at a particular intracellular location (e.g., the plasma membrane), it is possible to generate compartment-specific protein localization patterns using light (Figure 2A). This allows, for example, the localized recruitment of individual actin regulators at the cell cortex and the testing for how specific modifications in the molecular composition of actomyosin networks impact on cell shape changes. Alternative approaches include the use of photosensitive protein domains that block protein activity by sterically inhibiting interaction with endogenous binding partners (photo-caging) [10]. Upon illumination, unwinding of the photosensitive domain frees the target protein (photo-uncaging), allowing it to interact with its binding partners, thus restoring protein activity in a spatiotemporal controlled manner (Figure 2B). For example, direct photo-caging of Rho signaling components (e.g., RhoA, RhoGEF, and ROCK) or of specific signaling receptors that control actin dynamics, such as G-protein-coupled receptors, could be employed to increase contractility at will in defined cell populations. By modulating the power and the frequency of light pulses used to trigger optogenetic activation, it should be possible to address how actomyosin networks contract in response to inputs of different strength. In addition, optogenetics could be used to induce *de novo* assembly of actomyosin networks in noncontractile cells.

The feasibility of implementing optogenetics to modulate cell contractility and complex morphogenetic processes *in vivo* has been recently demonstrated in *Drosophila* [11]. During *Drosophila* gastrulation, the presumptive mesoderm is internalized through the formation of a groove called ventral furrow (VF) [12]. Upon apical accumulation of contractility complexes, ∼1000 ventral cells organized as a stripe along the embryonic anterior–posterior (a–p) axis constrict their apical surface, and invaginate as a group, folding into a tube [13]. Although there is compelling evidence that apical constriction facilitates tissue invagination 6, 14, it is not known whether contractile forces in ventral cells are needed throughout VF formation or whether they are necessary only to bend the tissue, whose internalization would be due to pushing forces exerted by lateral cells [15]. Another open question relates to how cells coordinate their contractile behavior to ensure a robust invagination. During *Drosophila* VF formation, cells constrict in an anisotropic fashion: they reduce their area along the dorsal–ventral (d–v) axis and remain elongated along the a–p axis [6]. This behavior has been explained with higher tension along the a–p axis compared to the d–v axis, which would cause cells to pull preferentially on their a–p neighbors. However, *in silico* models suggest that anisotropic constrictions might be the result of a gradient of contractility along the d–v axis, with cells closer to the ventral midline constricting more than cells farther away [15].

To address these questions, optogenetics was employed to inhibit phosphatidylinositol-4,5 bisphosphate [PI(4,5)P_2_] production at the plasma membrane during ventral furrow formation [11]. PI(4,5)P_2_ is of fundamental importance for the cortical recruitment of many actin-binding proteins [16], and therefore its acute depletion was expected to provide an efficient mean to inhibit apical constriction. To this end, the CRY2-CIB1 light-mediated protein heterodimerization system was used to control the plasma membrane recruitment of the catalytic domain of the inositol polyphosphate 5-phosphatase OCRL, which converts PI(4,5)P_2_ into phosphatidylinositol-4-phosphate [PI(4)P] [17]. Spatial accuracy was achieved using two-photon illumination that, by minimizing light scattering, allowed optogenetic activation with cellular precision on subminute timescales. Using this approach, it was possible to show that contractile forces generated in ventral cells are not only necessary to initiate tissue bending but are required throughout the invagination process. Moreover, the geometry in which cells are organized determines the emergence of anisotropic changes in their shape. This means that cells organized in a rectangular shape elongate along the longest side, while cells organized in a squared shape contract in a more symmetric fashion. An additional finding is that inhibiting apical constriction at suboptimal levels in a subgroup of ventral cells results in the stall of contractions in neighboring nonactivated cells [11]. Although the mechanisms underlying this phenomenon have not been clarified, it is tempting to speculate that it might be related to some form of **mechanotransduction**, which has been reported in both cell culture [18] and intact organisms during embryonic development 5, 19. During VF invagination, it has been shown that mechanical stimuli result in myosin accumulation at the apical cortex of cells, concomitant translocation of phosphorylated β-catenin in the nucleus, and subsequent expression of the mesoderm-specific transcription factor *twist*, which is required for ventral furrow invagination 20, 21. By modulating apical constriction in intact embryos, it should be now possible to study the dynamics of putative mechanosensing mechanisms. Moreover, optogenetic inhibition of cell contractility, in combination with *in toto* embryo imaging using selective plane illumination microscopy [22], should allow the investigation of the role played by extrinsic forces on VF formation, and of the impact of tissue tension on individual cell behavior.

The development of optogenetic methods to modulate cell contractility with subcellular precision will facilitate the study of other modes of epithelial remodeling, such as convergent extension, which is driven by contraction of specific interfaces in intercalating cells 23, 24, 25, 26, 27. Optogenetic strategies to achieve subcellular perturbations include the use of protein heterodimerization modules engineered with an anchor component that localizes to specific cellular compartments or subdomains of the plasma membrane (e.g., adherens junctions, lipid rafts). Such anchors would allow the localized recruitment of a protein of interest fused to a cognate light-sensitive component. Alternatively, the use of protein heterodimerization modules with fast (seconds) reversion kinetics [28] would ensure quick dissociation of molecules that diffuse away from the area of illumination. Another option is the use of the Phy–PIF protein heterodimerization system [29], which can be switched on or off at two different wavelengths. This would allow the generation of localized patterns of optogenetic activation by combining activation of a region of interest and deactivation of the surrounding area. The Phy–PIF system has recently proved an efficient means to control the subcellular localization of cell polarity components in zebrafish embryos [30]. In this study, an optogenetic engineered key component of the apical polarity complex, Pard3, could be reversibly localized to different plasma membrane regions, and its inheritance at cytokinesis controlled using pulses of red light. However, the Phy–PIF system requires the addition of an exogenous chromophore, which might limit its *in vivo* applications.

### Cell Migration

Locomotion is crucial for cells to move from one region to another and build organs during both embryonic development and homeostasis in adult organisms. Some cells, including leukocytes and hematopoietic stem cells, migrate as individuals [31]. Other cells migrate in a group, either as interconnected epithelial clusters or as **mesenchymal cells**, for example during gastrulation [32] and neural crest development [33]. In order to move in a specific direction, whether as individuals or as a group, cells have to acquire front/rear polarity. This polarity is established by directional cues, such as gradients of growth factors, chemokines, or extracellular matrix (ECM) components (reviewed in 34, 35).

Although the molecular players of cell migration are mostly known, it is less clear where and when their activity is needed to generate locomotion. It has been possible to start tackling this question by using photoactivatable derivatives of the small GTPase Rac1 (PA-Rac), which allow the modulation of Rac function at precise subcellular locations [10]. In cultured cells, activation of Rac1 at the cell edge was sufficient to promote membrane ruffling, recruit the Rac effector PAK, and direct cell movement. By contrast, deactivation of Rac1 led to membrane retraction at the site of irradiation, and stimulated ruffling in other areas of the cell. In addition, the use of PA-Rac1 made it possible to discover that myosin II is dispensable for the generation of Rac-induced protrusions, although it is necessary for cell movement [10]. This optogenetic tool was further exploited to test how Rac regulates the small GTPase RhoA, since it has been shown that Rac can both activate and repress RhoA 36, 37, 38. Photoactivation of Rac at defined subcellular locations led to the inhibition of Rho, whose activity was suppressed in Rac-induced protrusions [10]. However, in the context of normal motility both active Rac and RhoA are present at the leading edge, suggesting that inhibition of RhoA is either compartmentalized or kinetically controlled. *In vivo*, activation of Rac resulted in the formation of cellular protrusions and directed movement in both *Drosophila* border cell clusters and individual zebrafish neutrophils 39, 40. Furthermore, PA-Rac has been used to shed light on the function of Rac and PI3K during neutrophil migration. The most popular view is that PI3K promotes protrusion formation by stimulating Rac guanine nucleotide exchange factors (GEFs). However, in cells where PI3K was inhibited, the activation of Rac induced the formation of cellular protrusions, but it failed to rescue locomotion and front/rear polarity, suggesting that Rac and PI3K act through separate pathways to promote cell migration [40].

The migration of cell collectives shows many similarities with the migration of individual cells, however in this context cells maintain their apical–basal polarity and remain interconnected through cadherin-mediated adhesions. Moreover, front/rear polarity is established across the cell collective, and signaling is required only in ‘leading’ cells, which face the direction of migration [41]. Open questions relate to how groups of cells coordinate to move in the same direction, and which cues – chemical or mechanical – are transmitted by leading cells to trailing cells, and by surrounding cells and tissues to the collective. PA-Rac was used to address some of these questions during collective migration of *Drosophila* border cells*.* Changes in Rac activity in single cells affect the protrusive behavior of all the other cells in the cluster, and Rac activation in a single leading cell is sufficient to redirect the movement of the collective [39]. High levels of Rac in leading cells act as a directional cue by exerting on trailing cells a pulling force that is transmitted via E-cadherin-mediated contacts [42], and integrated through the JNK signaling pathway, the actin-binding protein moesin, and the small GTPase Rab11 39, 43.

Since optogenetics has proved a useful tool for uncovering the role of Rac GTPase during individual and collective cell migration, we expect that it should be possible to systematically dissect the spatial and temporal requirements of the known molecules involved in cell migration, at both the cellular and the tissue level. Currently, a few photoactivatable variants of Rho-family small **GTPases** are available: these optogenetic tools function either by promoting small GTPase oligomerization [44], or by activating or localizing GTPases or GTPase-specific GEFs to the plasma membrane 28, 29, 45, 46, 47, 48. These tools could allow us to understand where and when these proteins are required during cell locomotion. For example, it would be interesting to probe the role of RhoA in the assembly and disassembly of focal adhesions. Particularly promising, in this respect, is the development of a new system that allows the localized plasma membrane recruitment of RhoGEF and rapid activation of RhoA in cell culture [49]. Coupled to tension biosensors 6, 42, optogenetic tools that stimulate the activity of RhoA could help reveal the mechanisms through which tension regulates the formation of integrin-mediated contacts. Moreover, light-mediated control of PI3K localization, by enabling the manipulation of PIP_3_ levels 17, 50, might give insight into the requirement of this lipid for establishing and maintaining front/rear polarity. Finally, to gain a comprehensive understanding of cell polarity during migration, these tools could be complemented with other optogenetic systems to control organelle transport [38], activate proteins that promote actin polymerization such as diaphanous-related formins [51], and modulate the subcellular localization of specific polarity proteins [30].

Another outstanding question in cell migration is how cells ‘sense’ the environment to migrate in a specific direction. Current models suggest that prepatterned tracks of chemoattractants guide the movement of individual cells or collectives [35]. However, it has been shown that the sink activity of specific chemokine receptors is sufficient to generate a gradient of chemoattractants across the zebrafish lateral line primordium 52, 53, and that placodal cells contribute to the directional migration of adjacent neuronal crest cells [33]. Furthermore, optogenetic induction of Rac-mediated protrusions in neuronal crest cells is sufficient to promote cell separation and migratory behavior, further confirming that contacts with other cells and with the substrate can act as directional signals [54]. Further experiments using light-mediated perturbation of signaling inputs [55] and junctional remodeling (for example by controlling endocytosis, see later) will undoubtedly help to address how chemical and mechanical cues are integrated across individual cells and collectives.

### Cell–Cell Signaling

A key aspect of morphogenesis in multicellular organisms is that cells need to communicate with each other to coordinate their behavior. Fate determination, cell migration, cell proliferation, apoptosis, and changes in cell shape, adhesion, and polarity all arise in response to signaling cues. Despite their diversity, such cellular responses are regulated by a few conserved signaling pathways, including Wnt/Wingless, Hedgehog/Shh, Notch, EGFR, TGFβ, retinoic acid, and cytokine pathways. As there is no dedicated pathway for inducing each cell behavior, the outcome of signaling cues generally depends on a series of factors, such as signal strength and duration, and signal crosstalk, in addition to the transcriptional state of receiving cells [56].

Although there is a good understanding of the molecular players that regulate cell–cell communication, it is less clear how each player contributes to the generation of a specific cellular response. An intriguing question is how the spatial and temporal distribution of signals influences cell behavior. Where, when, and for how long is signaling required? Do signaling inputs need to be delivered constantly or rather at discrete frequencies? These questions, and how variations at specific points of signaling cascades affect cell behavior, remain hard to tackle using standard genetics, protein biochemistry, or chemical perturbations.

The application of optogenetics to the mitogen-activated protein kinase (MAPK/Erk) cascade has offered the opportunity to perturb this pathway with unprecedented temporal precision, and analyze the effect of such perturbation in a quantitative way. The MAPK/Erk signaling cascade is activated by different cues, and stimulates diverse responses, including cell proliferation and differentiation. Activated membrane receptors, such as receptor tyrosine kinases (RTKs) or integrins, recruit GEFs that activate the small GTPase Ras. Active Ras initiates the phosphorylation cascade composed of a MAPKKK (Raf), a MAPKK (MEK1/2), and MAPK (Erk). Activated Erk translocates to the nucleus where it regulates gene expression by phosphorylating a series of transcription factors. It is known that stimulating particular cell types with epidermal growth factor (EGF) results in transient Erk activation and cell proliferation, whereas nerve growth factor (NGF) drives sustained Erk activation and cell differentiation (reviewed in [57]). By exploiting a light-sensitive Ras activator, the MAPK/Erk pathway was found to respond differentially depending on the duration of the light stimulus [58]. This difference in the dynamics of MAPK/Erk activation has further been shown to regulate the proliferation versus differentiation decision in cultured cells [59]. Moreover, optogenetic activation of Ras showed that the frequency of the stimulus influenced the activation of the downstream transcription factor STAT3. STAT3 was activated only when the light stimulus was delivered for two hours. If the stimulus was delivered in two 1-hour blocks, it did not result in STAT3 activation [58]. These experiments demonstrate how optogenetics enabled the precise manipulation in time of signaling pathways to address key questions, such as how dynamic signals are transduced and which cell responses they control. Similarly, by locally activating PI(3,4,5)P_3_ signaling, the mechanisms driving the formation of growth-cone-like structures in mouse neurons have begun to be elucidated [60].

Optogenetic tools to achieve control over specific classes of RTKs and GPCRs have been also developed 61, 62, 63, thus offering the opportunity to gain quantitative control over individual signaling pathways. In addition, tools that allow the control of endocytosis with light, for example by inducing clathrin-light chain clustering [64], will enable the understanding of how this trafficking pathway regulates signaling activity. Indeed, receptor-mediated endocytosis can modulate signaling, by either internalizing the receptor from the plasma membrane, or by providing platforms (‘signaling endosomes’) that promote amplification of signals and crosstalk of pathways [65]. This is particularly relevant in *in vivo* contexts, for example during Notch signaling, a pathway responsible for a series of developmental events including somitogenesis, mesoderm induction, and neuronal development. The endocytic trafficking of Notch ligands Delta/Serrate/Lag2 in signal-sending cells is key for activating signaling in signal-receiving cells [66]. Knowing when, where – at the subcellular level – and for how long receptors, ligands, or ligand–receptor complexes have to be internalized will offer a more comprehensive understanding of how cell–cell signaling shapes developing organisms.

## Concluding Remarks

**Figure 3:**
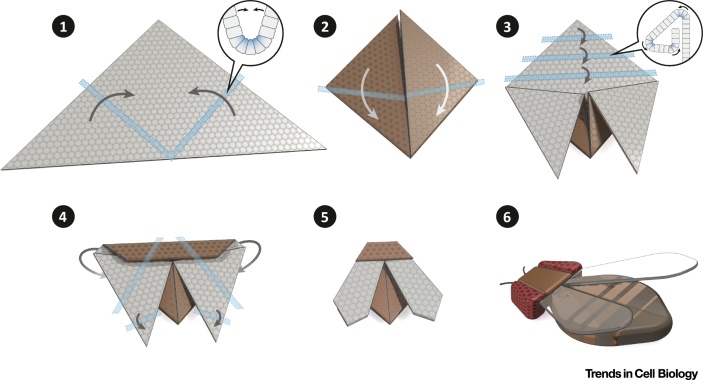
What Does it Take To Make it Fly? Synthetic Reconstitution of Morphogenetic Processes Using Optogenetic-Based Approaches. The case of the fly origami is intended to illustrate the fascinating question of the relationship between shape and function. An increasing number of studies suggest a link between three-dimensional tissue organization and cell differentiation 76, 77, 78. To what extent do changes in tissue shape/architecture impact on differentiation and organization of novel physiological functions during organismal development? Optogenetics provides a powerful new technique to address this fundamental question. In this visual model, a hypothetical 2D monolayer of progenitor cells is folded into a series of sequential 3D shapes by precise spatiotemporal modulation of cell behavior (e.g., contractility, relaxation, migration, apoptosis, etc.) using optogenetics (light patterns are represented as blue stripes). By monitoring the expression of differentiation markers or sensors of specific biochemical reactions, it should be possible to infer direct causal relationship between shape and function. Similar optogenetic approaches could also help to shape tissues of defined morphology for applications in regenerative medicine.

By employing light, optogenetics offers the possibility to simultaneously observe and perturb biological processes with subcellular resolution and on fast (seconds or minutes) timescales. In this review, we examined how the development and application of optogenetic tools have been instrumental to address some outstanding questions in cell and developmental biology. Importantly, as light can be administered in discrete pulses and at specific intensities and locations, optogenetics allows the quantitative perturbation of molecular processes in space and time. However, further optimization of optogenetic tools is needed especially for *in vivo* organismal applications. Collaboration between cell/developmental biologists, protein engineers, and chemists should focus on improving activation/reversion rates, and reducing the range of wavelengths to which the systems are responsive. In the future, it would be highly beneficial to combine the design of improved optogenetic tools with gene-editing techniques to generate light-sensitive alleles of key regulators of developmental processes. This should allow us to gain a quantitative understanding of biological systems, and include parameters such as frequency and intensity when describing signaling systems that orchestrate multicellular dynamics. Indeed, there is a growing need to dissect the circuits that regulate cell and tissue morphology, and uncover the contribution of each node in the development of a new shape. Several optogenetic approaches to modulate gene expression in cell culture and model organisms, including *Drosophila*, zebrafish, and mouse have been recently developed 45, 67, 68, 69, 70, 71, 72, 73. In combination with gene editing, these tools could be used to control gene expression endogenously. This is exemplified by work that combined transcription activator-like effector-based genome editing with the Cry2-CIB1 optogenetic system to control gene expression and modify histone marks [74]. This system, which was developed in mammalian cell culture and applied to living mice, can in principle be translated to other organisms, thus offering a new method for studying genetic and epigenetic regulation during animal development. Gaining a comprehensive understanding of how specific molecular and cellular activities regulate global morphological remodeling will be also instrumental for the field of ‘synthetic morphology’, a term coined by Davies [75] to indicate a subdiscipline of synthetic biology. The use of optogenetic approaches to reconstitute morphogenetic events in naïve cells and tissues will help us to understand the basic principles regulating development and the extent to which shape itself feeds back on developmental programs 76, 77, 78 (see Figure 3 and Outstanding Questions). In combination with computer simulations, optogenetics will provide a unique opportunity to test model predictions at all relevant spatial and temporal scales. Finally, it will potentially open new avenues for designing tissues and organs to be used in biotechnology and regenerative medicine.

Outstanding Questions What is the impact of geometrical constraints on individual cell behaviors and tissue/organ morphogenesis?

How does tissue tension influence morphogenetic movements? To what extent do mechanosensitive mechanisms coordinate group behavior?

What is the relationship between tissue architecture, cell differentiation, and distribution of signaling molecules?

How do neighboring tissues impact on each other's dynamics?

Given our current knowledge, would it be possible to reconstitute complex morphogenetic processes or embryoids from naïve cells that are reprogrammed and guided into three-dimensional shapes?

## Figures and Tables

**Table 1:** Commonly Used Photoreceptor Modules for Optogenetic Applications in Cell and Developmental Biology

Photosensitive Module	Heterodimerization/Protein Localization	Homodimerization	Oligomerization	Photo-caging	Comments
**PhyB**	PhyB/PIF6 29, 30, 58, 79, 80	N/A	N/A	N/A	Reversible with λ = 750 nm (dissociation time = ms), needs the cofactor PCB
**Cry2**	Cry2/CIB1 11, 17, 47, 59, 60, 62, 67, 69, 71, 74	N/A	Cry2olig [64] Cry2/Cry2 [44]	N/A	Reversible in the dark (dissociation time = min)
**LOV domains**	LOVpep/ePDZ 28, 38 FKF/GI 45, 73 iLID [46]	AuLOV [61] EL222 [70]	N/A	AsLOV2 10, 39, 51, 81 EL222 [70] iLID [46]	Reversible in the dark (dissociation time = s to h)

## Glossary

GTPases: enzymes that can bind and hydrolyze guanosine triphosphate (GTP). When bound to GTP, GTPases are active and can interact with their effectors. Binding to GTP is promoted by guanine nucleotide exchange factors (GEFs). The hydrolysis of GTP to GDP, which is triggered by GTPase-activating proteins (GAPs), switches GTPases off.
Mechanosensation/mechanotransduction: the ability of cells to sense and respond to mechanical stimuli. Cells sense the environment through cell–cell or cell–ECM contacts, and translate mechanical forces into biochemical signals. These signals can ultimately alter gene expression and, in turn, cell behavior.
Mesenchymal cells: arise from epithelial cells upon completing epithelial-to-mesenchymal transition (EMT). EMT results in the downregulation of cell–cell adhesions, the loss of apical–basal polarity, and the gain of migratory properties.
